# High-resolution freshwater dissolved calcium and pH data layers for Canada and the United States

**DOI:** 10.1038/s41597-024-03165-8

**Published:** 2024-04-11

**Authors:** Andrew J. Guerin, Andréa M. Weise, Jackson W. F. Chu, Mark A. Wilcox, Erin Sowerby Greene, Thomas W. Therriault

**Affiliations:** 1 Maurice Lamontagne Institute, Fisheries and Oceans Canada, 850 route de la mer, PO Box 1000, Mont Joli, Quebec G5H 3Z4 Canada; 2 Pacific Science Enterprise Centre, Fisheries and Oceans Canada, 4160 Marine Drive, West Vancouver, British Columbia V7V 1N6 Canada; 3 Pacific Biological Station, Fisheries and Oceans Canada, 3190 Hammond Bay Road, Nanaimo, British Columbia V9T 6N7 Canada

**Keywords:** Freshwater ecology, Limnology, Invasive species

## Abstract

Freshwater ecosystems are biologically important habitats that provide many ecosystem services. Calcium concentration and pH are two key variables that are linked to multiple chemical processes in these environments, influence the biology of organisms from diverse taxa, and can be important factors affecting the distribution of native and non-native species. However, it can be challenging to obtain high-resolution data for these variables at regional and national scales. To address this data gap, water quality data for lakes and rivers in Canada and the continental USA were compiled and used to generate high-resolution (10 × 10 km) interpolated raster layers, after comparing multiple spatial interpolation approaches. This is the first time that such data have been made available at this scale and resolution, providing a valuable resource for research, including projects evaluating risks from environmental change, pollution, and invasive species. This will aid the development of conservation and management strategies for these vital habitats.

## Background and Summary

Calcium concentration and pH are key determinants of many environmental and biological processes in freshwater ecosystems. Both variables regulate metabolic physiology in aquatic organisms, influencing reproduction, growth, and predator-prey interactions across a wide range of taxa including bacteria^1,2^, aquatic algae and diatoms^3,4^, molluscs^5^, crustacea^6^, and fish^4,7,8^. Since differences in these parameters can lead to detectable biological effects on individuals, populations, and communities^9,10^, pH and calcium concentration can both be important predictors of species distributions^11,12^ and are often used to evaluate the risk of establishment for invasive species, such as dreissenid mussels^13–15^. pH and dissolved calcium content of lakes influence their susceptibility to acidification^16,17^. They affect nutrient availability^18,19^, and play an important role in determining the environmental risks posed by metals and other contaminants by influencing their dissolution, mobilization, bioavailability, and toxicity^20–22^, as well as mediating their adsorption and desorption by microplastics^23,24^.

For large-scale studies at regional, national and continental levels, a common challenge facing freshwater researchers and resource managers is the availability of water quality data^17^, including calcium and pH. Such data are not readily available for all areas of North America, and given the large number of lakes, rivers, and other water bodies in Canada and the USA, measuring these variables at all sites would be prohibitively expensive and impractical. One way of improving water quality data coverage is to use existing measurements to predict values for unsampled locations *via* spatial interpolation^14,25,26^. This approach has several advantages: large amounts of data from multiple sources can be combined, no complex mechanistic modelling is required, and a range of established interpolation methods are available.

The goal of this work was therefore to use spatial interpolation to generate calcium and pH raster layers for the entirety of Canada and the continental USA at higher spatial resolution and coverage than previously available^13,15^. An expansive dataset covering Canada and the USA (1,347,887 calcium measurements from 97,648 locations, and 8,789,005 pH measurements from 208,784 locations) was compiled from multiple governmental, non-governmental, and academic sources, and used to generate spatially interpolated maps of these variables at a 10 × 10 km resolution. These layers will be of value for projects requiring calcium and pH data at regional to continental scales, including understanding past and present sensitivity of lakes and rivers to acidification^16,17^, assessment of regional variation in the risks posed by contaminants^20^, ecological niche modelling^27^, and invasive species risk assessment^13,15^.

## Methods

### Data sources

Since Canada lacks a centralised repository for water quality data, georeferenced Canadian water quality records were obtained from multiple sources: publicly-accessible federal^28–31^, provincial and territorial agency databases^32–40^; non-governmental open access data repositories - the Atlantic Datastream (https://atlanticdatastream.ca/)^41–93^ and the Mackenzie Datastream (https://mackenziedatastream.ca/)^52,94–115^; published reports and primary literature^116–124^; a previous invasive species risk assessment^15^; and directly from contacts in relevant agencies in each of the provinces and territories (Table 1). Records for the United States (including Alaska, but excluding Hawaii) were obtained from the Water Quality Portal^125^, which combines data from federal, state, tribal and local agencies; the *dataRetrieval* package^126^ was used to directly download data for sites with calcium and pH data collected between 2000 and 2021 (Water Quality Portal accessed 15^th^ February 2021). To ensure that records were as contemporary as possible while retaining high spatial coverage, records from before 2000 were excluded for most sources. However, older records were retained for some areas of Canada (particularly the Territories) where fewer data were generally available. All data handling, processing and interpolation was conducted in R v4.1.0^127^.

**Table 1:** Summary of data sources used.

Type	Source	Spatial coverage	Temporal coverage	n Sites	
				Ca	pH
FAD	Environment and Climate Change Canada^28^	Canada, AK	2000–2019	282	279
FAD	Environment and Climate Change Canada^29^	ON	2000–2018	4172	4357
FAD	Environment and Climate Change Canada^30^	Canada	2002–2018	—	267
FAD	USGS^31^	AK,YT	2009–2014	85	92
PDR	Atlantic DataStream^41–93^	NB,NL,NS,PE	2000–2020	555	2214
PDR	Mackenzie DataStream^52,94–115^	AB,BC,NT,YT	2000–2018	480	707
PDR	Water Quality Portal^125^	USA	2000–2021	76013	188951
PTA‡	Government of Alberta^32^	AB	2000–2020	254	302
PTA	Government of British Columbia^33^	BC	2000–2020	2903	3033
PTA†	Government of Manitoba	MB	2011–2018	262	302
PTA	Government of New Brunswick^34^	NB	2000–2020	635	663
PTA	Government of Newfoundland and Labrador^35^	NL	2019–2020	—	29
PTA	Government of Nova Scotia^36^	NS	2002–2017	—	5
PTA†	Government of the Northwest Territories	NT	1982–2021	97	97
PTA†	Natural Resources and Forestry Ontario	ON	2008–2017	1327	1346
PTA	Environment, Conservation and Parks Ontario^37^	ON	2000–2019	541	594
PTA	Government of Prince Edward Island^38^	PE	2001–2020	72	219
PTA‡	Gouvernement du Québec^39^	QC	2005–2020	1065	652
PTA‡	Water Security Agency, Saskatchewan^40^	SK	2010–2020	682	476
PTA†	Government of Yukon, Environment Department	YT	2010–2021	325	343
PRP	Antoniades *et al*.^116,117^	NT,NU	1996–2000	86	86
PRP	Filazzola *et al*.^118^	North of 50°N	1998–2016	—	148
PRP	Joynt and Wolfe^119^	NU	1995	—	56
PRP	Michelutti *et al*.^120,121^	NT,NU	1997–1998	37	37
PRP	Pienitz *et al*.^123^	NT	1991	24	24
PRP	Ruhland *et al*.^124^	NT,NU	1996,1998	53	53
OT	2012 Risk Assessment^15^, 2004 Acid Deposition Assessment^122^	AB,BC,MB,NB, NL,NS,ON,QC, SK	1983–2011	8703	4088
OT†	Conseil de gouvernance de l’eau des bassins versant de la rivière Saint-François (and partners)	QC	2018–2020	116	418
OT†	Kivalliq Inuit Association, Crown-Indigenous Relations and Northern Affairs Canada	NU	2004–2020	56	54

FAD = Federal Agency Database (Canada/US); PDR = Public Data Repository; PTA = Provincial or Territorial Agency (Canada); PRP = Peer-Reviewed Publication; OT = Other Organisation or Data Source. ^†^ data provided directly by agency contacts, not publicly accessible; ^‡^ includes data provided by agency contacts and publicly accessible agency databases; all other data were obtained from publicly accessible sources.

### Data processing and preparation for interpolation

Records from appropriate site types (lakes, rivers, ponds, and streams) were selected where possible, although most data sources did not provide this information. Records from marine waters or in proximity to mines, industrial facilities, wastewater treatment infrastructure or other potentially-contaminated sites were excluded if this information was provided. For USA Water Quality Portal data, for example, this was done by excluding records with certain keywords (e.g. “WASTEWATER”) in the site name or site description fields. Records with various map datums (NAD27, NAD83, WGS84) were included without correction; differences among these three major datums are generally less than a few hundred meters, which is an acceptable degree of positional error given the intended final resolution of the interpolated data layers. In any case, most records did not include map datum information, although records which specified unusual or unrecognised map datums were excluded. Data were inspected for clearly incorrect positions (e.g., points plotting outside of the relevant state, province, territory, or points plotting in the ocean); these were corrected where possible. Records that lacked critical metadata (i.e., coordinates, date, etc.), had obvious position or date errors that could not be easily rectified, were flagged at the source with quality control concerns, or had impossible (e.g., negative) measurements, were excluded.

‘Total’ and ‘Dissolved’ calcium were the most commonly recorded fractions, but data for other fractions were sometimes provided. Analysis of data from samples where more than one fraction was measured demonstrated strong positive correlations with slope close to 1 among the most commonly measured fractions (Table 2). Consequently, where data for multiple fractions were provided, measurements of ‘Dissolved’ calcium were preferred, but most other fractions were treated as equivalent and used where ‘Dissolved’ data were not provided. Other fractions were occasionally provided, including ‘Filterable’ and ‘Fixed’ calcium; insufficient data were available to compare these with ‘Dissolved’ calcium, and since they were extremely rarely encountered, they were excluded. In any case, large numbers of records did not provide information on the fraction analysed; their removal would have had a highly detrimental impact on the extent of the available data, so they were retained and assumed to be equivalent to ‘Dissolved’. Calcium concentrations were converted to consistent units (mg L^−1^) and records without units (<0.01% of records) were excluded.

**Table 2:** Comparison of “Dissolved” calcium versus different calcium fractions measured for samples where measurements for more than one fraction were supplied in the source data.

Comparison	n	ρ	*p*-value	Slope
“Dissolved” vs “Total”	139453	0.977	<0.001	1.019
“Dissolved” vs “Total Recoverable”	17791	0.982	<0.001	1.019
“Dissolved” vs “Recoverable”	9513	0.967	<0.001	1.023
“Dissolved” vs “Extractable”	490	0.947	<0.001	0.969
“Dissolved” vs “Soluble”	267	0.977	<0.001	0.998

ρ = Pearson’s correlation coefficient.

Some records had extremely high calcium concentrations, including some well over 1000 mg L^−1^; these values were generally considered unfeasible, as freshwater calcium concentrations rarely exceed 450 mg L^−1^ and are typically much lower^128^. Anomalously high calcium concentrations may result from inclusion of inappropriate sample types (e.g., contaminated water, industrial effluents, marine samples), equipment malfunction, and data entry errors. A cut-off of 500 mg L^−1^ was therefore set and all records with higher calcium concentrations were excluded; this represented <0.2% of all records. The only exceptions to this rule were samples from the Pecos and Wichita River systems in Texas; calcium concentrations above 500 mg L^−1^ are not unusual in this area^129,130^, and removing all such records left a notable gap in spatial coverage in an area with already sparse data coverage. Instead, all records above 500 mg L^−1^ for this area were set to 500 mg L^−1^ to maintain consistency with the rest of the data, while avoiding the loss of spatial coverage. For records with calcium concentrations below 0.05 mg L^−1^ (a common detection limit), one of two approaches was taken. Where records were flagged as being ‘below detection limit’, or where an explicit detection limit was given for values below 0.05 mg L^−1^, records were set to 0.05 mg L^−1^ for consistency across the dataset (<0.005% of all records). Other records with calcium concentrations less than 0.05 mg L^−1^ were excluded (<0.05% of all records). For pH data, records with values lower than 2.5 or above 12.5 were excluded, although for most sources all records fell within this range.

Duplicate data (duplicate records present in individual data sources, presence of the same data in multiple sources) and pseudo-duplicate data (lab and field replicates, samples collected simultaneously from different depths at a location) were handled by calculating an average (median) using all records for each site on each date. For each variable, these site-date medians were then used to calculate the following summary statistics for each site across all dates: mean, standard deviation, 25^th^ percentile, 50^th^ percentile (median), 75^th^ percentile, minimum, maximum (all of these summary statistics are included in the shared databases, see Data Records section). For spatial interpolation, the median value for each site was used, since this measure is comparatively robust to outliers. Medians for each site were converted to spatial data and reprojected into the North America Albers Equal Area Conic projection, using the *sf* package^131^.

### Spatial interpolation methods

To select the approach used to generate the interpolated data layers, three interpolation methods were compared (Table 3): nearest neighbour (NN), inverse distance weighting (IDW), and ordinary kriging (OK). NN is the simplest method, providing a baseline against which the more advanced methods can be compared; each point for which an interpolated value was required was assigned the value from the closest available data point. IDW uses a combination of values from multiple data points, weighted by distance. For IDW, arbitrary or ‘standard’ values for *nmax* (the maximum number of points to be considered when predicting a value for a specific grid cell) and *idp* (the inverse distance power parameter, which controls how the weighting of data points varies with distance) are often used^132^. In this case, however, the *optim* function was used to find values of *idp* and *nmax* for the calcium and pH data which minimised two different error metrics, root-mean-square error (RMSE) and mean absolute error (MAE), during preliminary 5-fold cross-validation (Table 4). OK is a geostatistical technique, which uses a fitted model of the spatial autocorrelation among data points (a ‘semi-variogram’ or ‘variogram’) to derive the weights used for the interpolation of values to each grid cell. OK often generates superior results to IDW^133^, but this is not always the case^132,134^. An additional advantage of OK is that it generates a measure of statistical uncertainty (Kriging variance) for each interpolated value; this is not typically provided by other methods. Kriging variance is influenced by the distances from the interpolated points to locations with data, and by the spatial covariance relationship determined by the fitted variogram; greater variance indicates greater distance from measured values and thus greater uncertainty in the interpolated values. Variograms for each variable were fitted using the *automap* package^135^, which automatically selects relevant models and parameter values that best fit the empirical variogram (Table 3), although constraints can be applied to the process. In this case, variograms were fitted with and without a fixed ‘nugget’ of zero, since manual setting of this parameter can sometimes be advantageous^136^. In all cases, OK was restricted to a *nmax* of 100 and *nmin* (the minimum number of data points to consider) of 15; changes to these numbers had little to no impact on the error metrics obtained during preliminary 5-fold cross-validation. Spatial models for all interpolation methods were fit using the *gstat* package^137^.

**Table 3:** List of Interpolation methods used, including parameter values (where applicable).

Interpolation Method	Calcium	pH
**Nearest Neighbour (NN)**	NN interpolation uses only data from nearest point (*nmax* = 1)	
Inverse Distance Weighting, Optimsed for RMSE (IDW-OR)	*nmax* = 15, *idp* = 1.2	*nmax* = 19, *idp* = 1
Inverse Distance Weighting, Optimsed for MAE (IDW-OM)	*nmax* = 14, *idp* = 2.2	*nmax* = 16, *idp* = 1.1
**Ordinary Kriging (OK)**	Model: Matern/Stein	Model: Matern/Stein
	Nugget: 700	Nugget: 0.4
	Sill: 3413	Sill: 0.86
**Ordinary Kriging with zero nugget (OK-ZN)**	Model: Matern/Stein	Model: Matern/Stein
	Sill: 2076	Sill: 0.92

**Table 4:** Error metrics considered for comparison of interpolation methods.

Metric	Formula	Interpretation
Correlation coefficient, *r*	Correlation between ${Z}_{i}^{{\prime} }$ and *Z*_*i*_	High correlation between observed and interpolated values suggests accurate interpolation
Mean Absolute Error (MAE)	$\frac{{\sum }_{i=1}^{n}| {Z}_{i}^{{\prime} }-{Z}_{i}| }{n}$	A measure of the average magnitude of prediction errors
Root Mean Square Error (RMSE)	$\sqrt{\frac{{\sum }_{i=1}^{n}{({Z}_{i}^{{\prime} }-{Z}_{i})}^{2}}{n}}$	Another measure of the average prediction error. Compared to MAE, RMSE is more sensitive to large outliers.
Mean Bias Error (MBE)	$\frac{{\sum }_{i=1}^{n}{Z}_{i}^{{\prime} }-{Z}_{i}}{n}$	By including the sign of individual residuals, MBE shows whether (on average) the interpolation tends to over- or under-predict
Median Symmetric Accuracy (MSA)	$exp(Median({|\log }_{e}({Z}_{i}/{Z}_{i}^{{\prime} })|))-1$	Measure of ‘typical’ proportional error, symmetric and robust to outliers

*Z*_*i*_ is the observed value at a point, ${Z}_{i}^{{\prime} }$ is the value interpolated for that point during LOOCV.

Leave-one-out cross-validation (LOOCV) was performed for each method to compare their predictive accuracies. This technique drops an individual point from the dataset and then uses the remaining data to interpolate a value for the location of the dropped data; this is repeated for all available data points. The interpolated values for each point were compared to the real measured values and used to calculate multiple performance metrics (Table 4): the correlation coefficient *r*; three absolute error measures, RMSE, MAE, and the mean bias error (MBE); and a measure of relative error, the median symmetric accuracy^138^ (MSA). The interpolation methods were compared by considering their scores in these metrics. Initially, the intention was to use an ‘objective’ function^136^ to integrate these metrics into a single performance score. However, this was not necessary, since for both calcium concentration and pH one method had the best scores in all key metrics (see Technical Validation). Since predictive accuracy of any interpolation method can vary spatially^132,136^, error metrics were also calculated using data from each individual province, territory and state.

On the basis of these comparisons a final interpolation method was selected for each variable. Calcium and pH values were then interpolated onto a grid with a cell size of 10 × 10 km^2^ using the *gstat::predict* function and the resulting grids were converted to raster format^139^. Interpolated rasters were masked using outlines of Canada and the USA from the *rnaturalearth* package^140^. Rasters of Kriging variance for each variable were also generated at the same resolution.

## Data Records

Project data are available at Data Dryad^141^. The data provided include the final interpolated rasters (and kriging variance rasters) for calcium and pH, the point data used for the interpolations (summary statistics for each site) and the underlying data for each site on each date (Table 5).

**Table 5:** List of data provided in the associated data repository^141^.

	File name	Description
Rasters: Ca	calcium-KR-97648-median_10 km_ZN_masked.tif	interpolation, masked
	calcium-KR-97648-median_10 km_ZN_variance_masked.tif	kriging variance, masked
	calcium-KR-97648-median-10km-ZN.tif	interpolation, unmasked
	calcium-KR-97648-median-10km-ZN_variance.tif	kriging variance, unmasked
Rasters: pH	ph-KR-208784-median_10km_ZN_masked.tif	interpolation, masked
	ph-KR-208784-median_10km_ZN_variance_masked.tif	kriging variance, masked
	ph-KR-208784-median_10km_ZN.tif	interpolation, unmasked
	ph-KR-208784-median_10km_ZN_variance.tif	kriging variance, unmasked
Data: Ca	sites_calcium_85747.csv	site summary data
	metadata_sites_calcium_85747.csv	
	site-date_calcium_985955.csv	summary data for all dates with records at all sites
	metadata_site-date_calcium_985955.csv	
Data: pH	sites_ph_201183.csv	site summary data
	metadata_sites_ph_201183.csv	
	site-date_ph_3525217.csv	summary data for all dates with records at all sites
	metadata_site-date_ph_3525217.csv	
Other	source_ids.csv	list of data source IDs

Masked rasters have been masked using country outlines such that values are only given for land area, and are therefore ‘ready-to-use’. Note that databases (.csv files) only include data from shareable (open source) providers.

Rasters were generated by Ordinary Kriging with a pre-defined zero nugget: for both variables this was the best-performing method (see Technical Validation). All rasters use the North America Albers Equal Area projection (ESRI:102008), have a resolution of 10 × 10 km, and have been provided in geotiff format. For each interpolation, the associated kriging variance rasters have been provided; these can be used to identify areas of higher uncertainty resulting from low availability of water quality data. Rasters are provided both ‘masked’ (using country outlines for the USA and Canada from the *rnaturalearth* package^140^ such that values are only provided for land area) and ‘unmasked’. The latter allow users to use their own territorial outlines for masking, to resample the rasters at different resolutions, or to reproject the rasters (for example into a latitude-longitude projection). It is advisable to perform these latter operations prior to masking the rasters with territory outlines. Some example R scripts to facilitate masking and reprojection can be found in the associated GitHub repository^142^ (see Code Availability, below).

The ‘sites’ files contain the site data used for the interpolations (Table 5). This includes the following summary statistics for each site: median (used for the interpolations), number of dates with data, total number of records included, mean, standard deviation, minimum, maximum, 25^th^ percentile, 75^th^ percentile. Information on data sources and years with data is also included. The ‘site-date’ files contain summary data for each site on each date with available data: the median, mean, standard deviation, minimum, maximum, 25^th^ percentile, 75^th^ percentile, and number of records. Data sharing agreements with some organisations do not permit open sharing of their data and thus these records are not included in the databases (11,901 sites for calcium, 7601 sites for pH). For a small number of sites for which both public and proprietary data were available (calcium: 383 sites, pH: 61 sites), summary statistics have been recalculated using only public data; consequently, the values provided may not exactly match those used for the interpolations. The associated metadata files include full information on the contents of each of the data files. Finally, the source_ids.csv file includes identifying information for the data sources included in the databases.

**Fig. 1:**
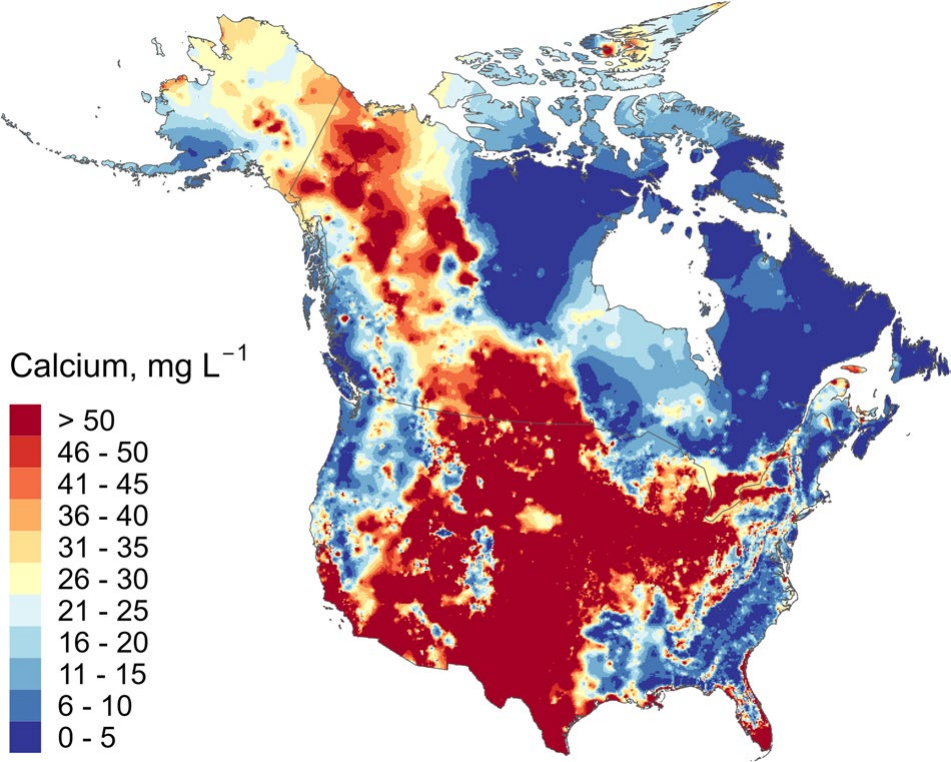
Interpolated freshwater calcium concentration map for Canada and the USA, generated using zero-nugget Kriging interpolation.

**Fig. 2:**
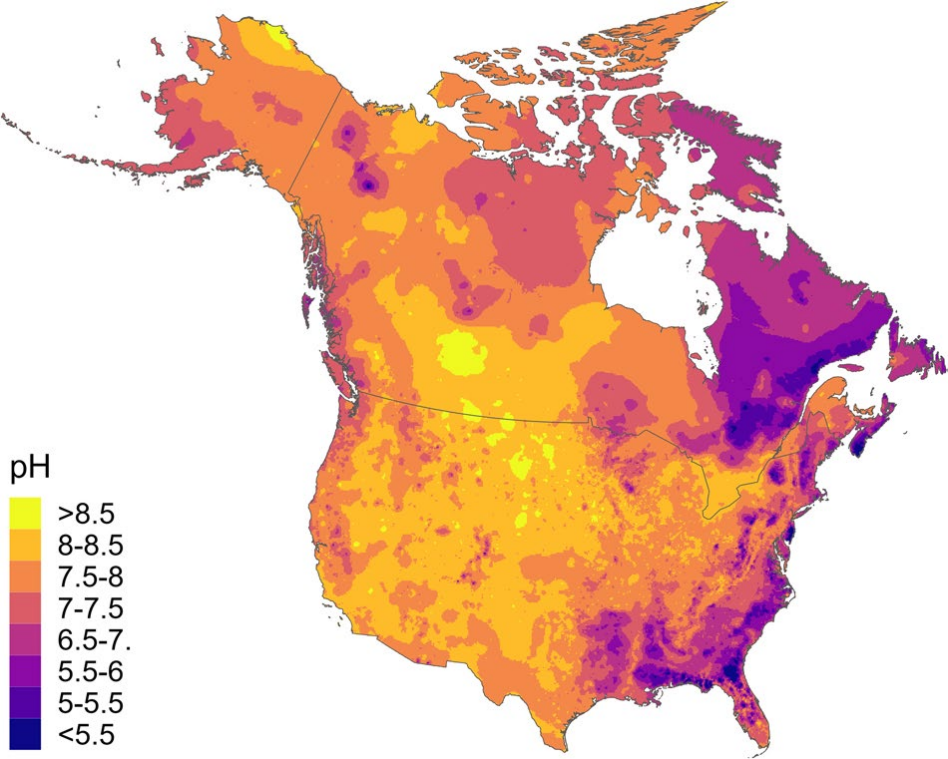
Interpolated freshwater pH map for Canada and the USA, generated using zero-nugget Kriging interpolation.

There are obvious spatial patterns in freshwater calcium concentrations across the continent (Fig. 1), reflecting the relationship with the chemical composition of the underlying bedrock. These include large areas of comparatively low calcium on the east and west coasts of Canada and the USA, and a large area corresponding to the Canadian Shield geological region. Calcium concentrations are comparatively high (30 mg L^−1^ or greater) across a continuous broad area running from the southern United States up to Yukon and Alaska. Areas of high and low calcium tend to correspond with areas of high and low pH (Fig. 2).

## Technical Validation

### Calcium

**Fig. 3:**
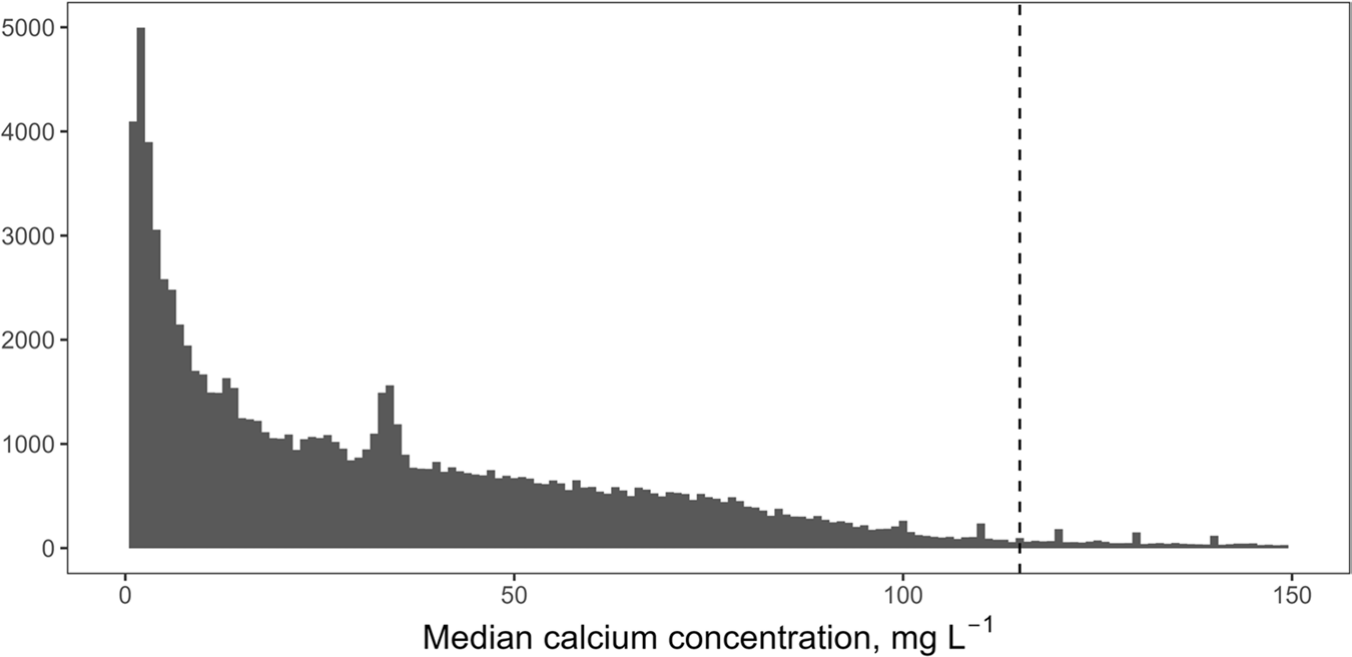
Frequency distribution of site median calcium concentrations. Dashed line marks the 95^th^ percentile. X-axis is truncated at 150 mg L^−1^; a small proportion of sites (~3%) had higher values.

**Fig. 4:**
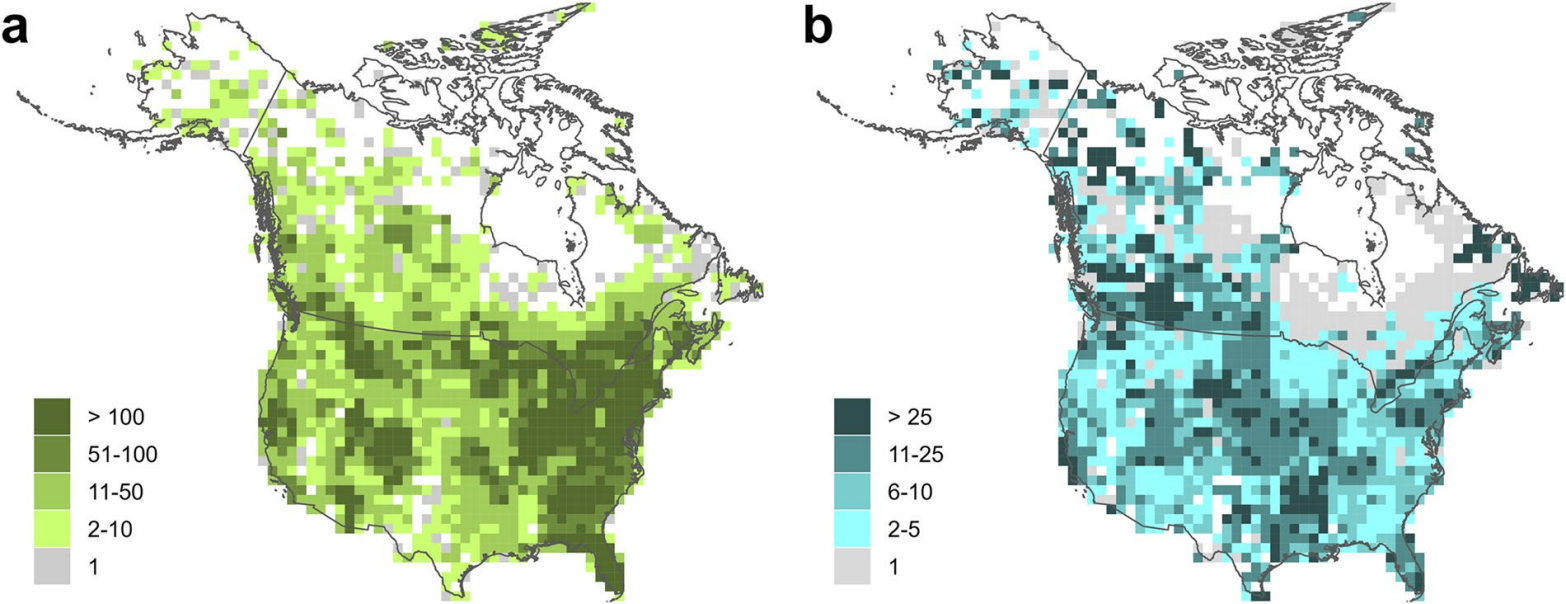
Calcium concentration data coverage. (**a**) Sites per 10,000 km^2^ (**b**) Sampling intensity (mean dates sampled per site per 10,000 km^2^).

**Fig. 5:**
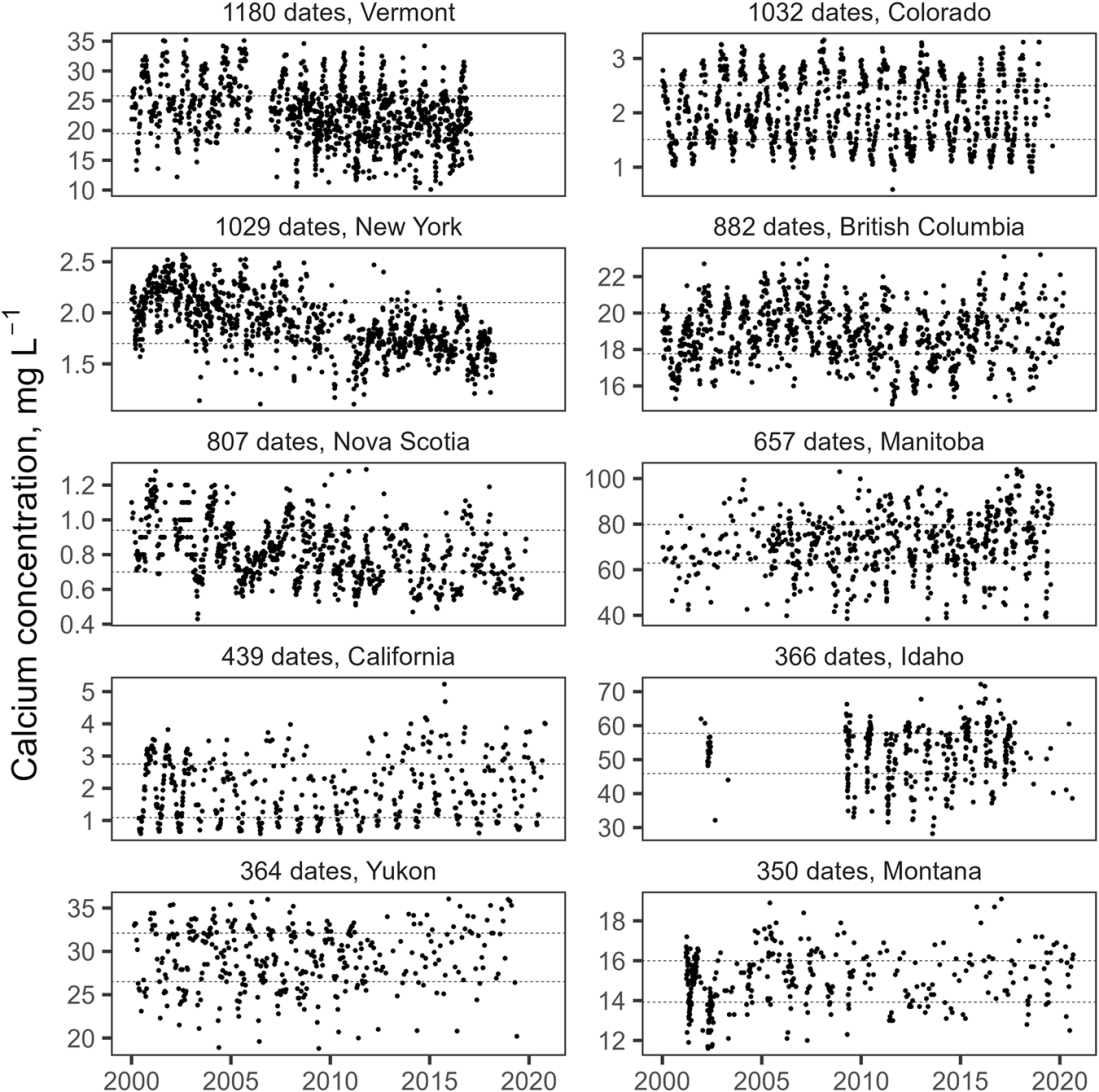
Temporal variation in dissolved calcium concentration for ten sites, selected (from among the 100 sites with data for the most dates) to have the longest temporal coverage and to come from 10 different administrative regions; source region given for each plot, along with number of dates with data. Seasonal fluctations are evident for most of the sites, but are small compared to spatial variation across Canada and the USA. Dotted lines mark the interquartile range for each site. Outliers have been removed for presentation, but were not excluded from calculation of site statistics. Note the differences in vertical scales for individual plots.

The final calcium database used for the interpolations included records for 97,648 sites; the publicly shareable dataset includes 85,747 sites. Median calcium concentrations for individual sites ranged from 0.06 to 500 mg L^−1^, but 95% of sites had median calcium concentrations of 115 mg L^−1^ or lower (Fig. 3). The highest concentrations of sites were mostly in the eastern United States and parts of southern Ontario, Quebec, and New Brunswick, while coverage was lowest in Alaska, the Canadian territories (Yukon, Northwest Territories, and Nunavut), and parts of northern Quebec (Fig. 4a). The majority of sites (56%) were sampled multiple times; individual sites were sampled from 1 to over 1000 times (median dates sampled = 2, mean dates sampled = 10.6). In most areas sites were, on average, sampled at least twice; however, there were areas of northern Ontario and Quebec where only a single data point was provided for most sites (Fig. 4b). Some of the provided data for this area were already temporally-averaged values, so this does not mean that all data points for these areas were based on single measurements. Temporal variation in measurements from individual sites is to be expected as a result of measurement error and temporal change, such as seasonal fluctuations in calcium concentrations (Fig. 5). However, the scale of temporal variation at individual sites was generally smaller than the spatial variation among sites. The interquartile range for temporally-averaged calcium concentrations across all sites was 48.5 mg L^−1^, while the median interquartile range for calcium measurements at individual sites was 5 mg L^−1^, and 75% of sites had an interquartile range of 12.5 or less.

For the interpolation of calcium concentrations, the zero-nugget Kriging method (OK-ZN) had the highest *r* value and the lowest error metrics (excluding the proportional error, MSA, which was very similar to the lowest value); in particular, the bias error (MBE) was lower than the other methods (Table 6). The IDW interpolations, however, were not substantially worse. At the province, territory, and state level, the outcome was mostly similar: OK-ZN was the best or joint-best method in 53 out of 62 cases (Tables 7, 8); OK was slightly superior for four areas, the IDW methods (IDW-OR and IDW-OM) were superior for 4 areas, and in one US state NN was the best method. There appeared to be no tendency for the best approach to vary with number of data points in each area; OK-ZN was generally superior for states, provinces and territories with low (e.g., Mississippi, n = 75) and high (e.g., Florida, n = 13,591) numbers of data points. Consequently, the zero-nugget kriging interpolation (OK-ZN) was selected as the preferred interpolation method.

**Table 6:** Error Metrics for calcium interpolation methods, generated via leave-one-out cross-validation.

Method	*r*	RMSE	MAE	MBE	MSA
NN	0.78	32.77	13.25	0.09	**0.21**
IDW-OR	0.82	28.03	12.25	0.16	0.23
IDW-OM	0.82	28.63	12.09	0.13	**0.21**
OK	0.82	28.61	13.39	**−0.01**	0.30
OK-ZN	**0.84**	**27.06**	**11.79**	**0.01**	0.22

The best interpolation methods for each metric are highlighted in bold. Metrics: *r* = correlation coefficient, RMSE = Root Mean Square Error, MAE = Mean Absolute Error, MBE = Mean Bias Error, MSA = Median Symmetric Accuracy. Methods: NN = nearest neighbour; IDW-OR = inverse distance weighting, RMSE-optimised; IDW-OM = inverse distance weighting, MAE-optimised; OK = ordinary kriging, unconstrained nugget; OK-ZN = ordinary kriging, zero nugget.

**Table 7:** Best-performing calcium and pH interpolation methods for each Province / Territory (Canada), according to LOOCV error score and correlation between observed and predicted values.

Province / Territory	Calcium		pH	
	n	Best	n	Best
Alberta	841	IDW-OR (OK-ZN)	475	IDW-OM
British Columbia	3384	OK-ZN	3154	OK-ZN
Manitoba	899	OK-ZN	814	OK-ZN (IDW-OM)
New Brunswick	936	OK-ZN	1221	OK-ZN
Newfoundland and Labrador	117	OK-ZN	330	OK-ZN
Nova Scotia	270	OK-ZN	657	IDW-OM
Northwest Territories	428	OK-ZN	504	IDW-OR/IDW-OM
Nunavut	164	OK	313	OK-ZN
Ontario	7819	OK-ZN	6537	OK-ZN
Prince Edward Island	75	OK-ZN	478	OK-ZN
Quebec	4619	OK-ZN	4259	OK-ZN
Saskatchewan	1657	OK-ZN	630	OK-ZN
Yukon Territory	365	OK-ZN	398	IDW-OM

n = number of sites. Methods: NN = Nearest Neighbour; IDW-OR = Inverse distance weighting, RMSE-optimised; IDW-OM = Inverse distance weighting, MAE-optimised; OK = Kriging; OK-ZN = Kriging, zero nugget. Where two methods are presented separated by ‘/’ this indicates that the two methods performed equally well. Methods presented in parentheses were those with the highest correlation coefficient, r, in cases where this was not the same method with the best scores in the other error metrics.

**Table 8:** Best-performing calcium and pH interpolations for each State (USA).

State	Calcium		pH	
	n	Best	n	Best
Alaska	396	OK-ZN	1020	OK-ZN
Alabama	1274	OK-ZN	2635	IDW-OM/OK-ZN
Arkansas	848	OK-ZN	1258	IDW-OM
Arizona	2102	OK-ZN	3416	IDW-OM
California	2904	OK-ZN	6451	OK-ZN
Colorado	4355	OK-ZN	6296	OK-ZN
Connecticut	78	NN	168	IDW-OM (NN)
Delaware	75	OK-ZN	559	OK-ZN
Florida	13591	OK-ZN	29471	OK-ZN
Georgia	1954	IDW-OM	3554	OK-ZN (IDW-OM)
Iowa	266	OK	5001	OK-ZN
Idaho	591	OK-ZN	1322	OK-ZN
Illinois	2129	OK-ZN	3406	OK-ZN
Indiana	2260	OK-ZN	8845	IDW-OM (OK-ZN)
Kansas	1198	OK-ZN	1045	OK-ZN
Kentucky	751	OK-ZN	1394	IDW-OM (OK-ZN)
Louisiana	357	OK	935	IDW-OM (OK-ZN)
Massachusetts	344	OK-ZN	1442	OK-ZN
Maryland	579	OK	2942	OK-ZN
Maine	262	OK-ZN	1506	OK-ZN
Michigan	2367	OK-ZN	3046	IDW-OM
Minnesota	1994	OK-ZN	14282	OK-ZN
Missouri	847	OK-ZN	2975	OK-ZN
Mississippi	73	OK-ZN	1408	OK-ZN
Montana	4081	OK-ZN	5860	OK-ZN
North Carolina	938	OK/OK-ZN	2148	OK-ZN
North Dakota	1321	OK-ZN	1856	IDW-OM/OK-ZN
Nebraska	265	OK-ZN	1368	OK-ZN (IDW-OM)
New Hampshire	324	OK-ZN	2792	OK-ZN
New Jersey	1090	OK-ZN	4108	IDW-OM
New Mexico	1100	OK-ZN	1491	OK-ZN
Nevada	836	OK-ZN	1816	OK-ZN
New York	1819	OK-ZN	2611	OK-ZN
Ohio	5836	OK-ZN	6159	OK-ZN
Oklahoma	402	OK-ZN	4261	OK-ZN (IDW-OR)
Oregon	855	OK-ZN	3750	OK-ZN
Pennsylvania	1831	OK-ZN	2927	IDW-OM (OK-ZN)
Rhode Island	26	IDW-OM	59	IDW-OM
South Carolina	1509	IDW-OR	1958	OK-ZN
South Dakota	497	OK-ZN	3200	OK-ZN
Tennessee	795	OK-ZN	5811	OK-ZN
Texas	528	OK-ZN	5077	OK-ZN
Utah	2554	OK-ZN	3641	OK-ZN
Virginia	1665	OK-ZN	8271	OK-ZN
Vermont	1893	OK-ZN	2169	IDW-OM (OK-ZN)
Washington	335	OK-ZN	1876	IDW-OM/OK-ZN
Wisconsin	1952	OK-ZN	7308	OK-ZN
West Virginia	1339	OK-ZN	2553	OK-ZN
Wyoming	684	OK-ZN	1567	OK-ZN

See Table 7 for abbreviations.

### pH

**Fig. 6:**
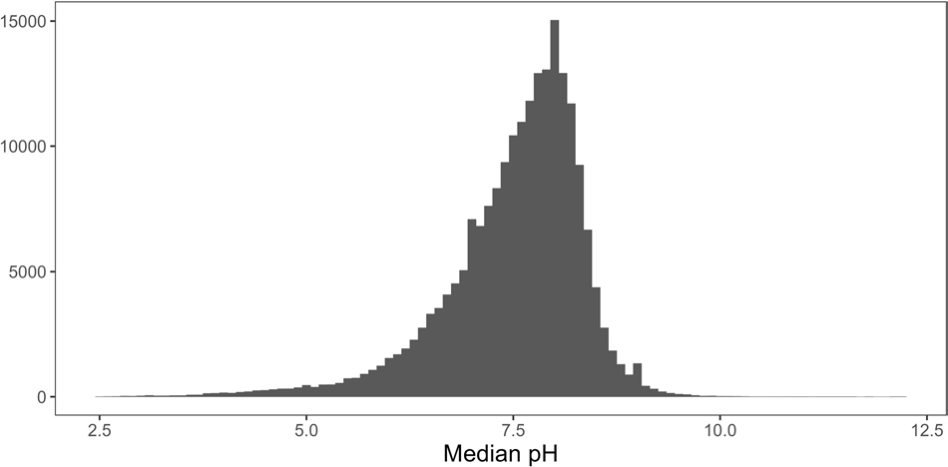
Frequency distribution of site median pH values.

**Fig. 7:**
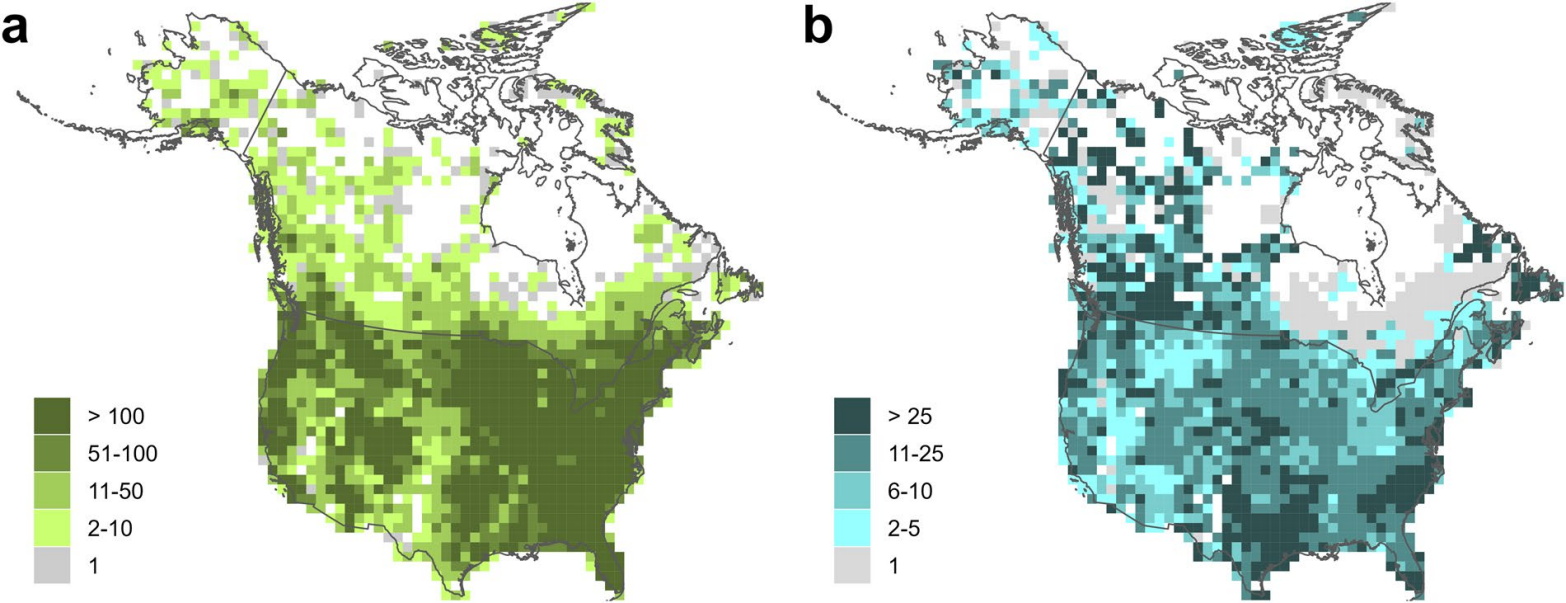
pH data coverage. (**a**) Sites per 10,000 km^2^ (**b**) Sampling intensity (mean dates sampled per site per 10,000 km^2^).

**Fig. 8:**
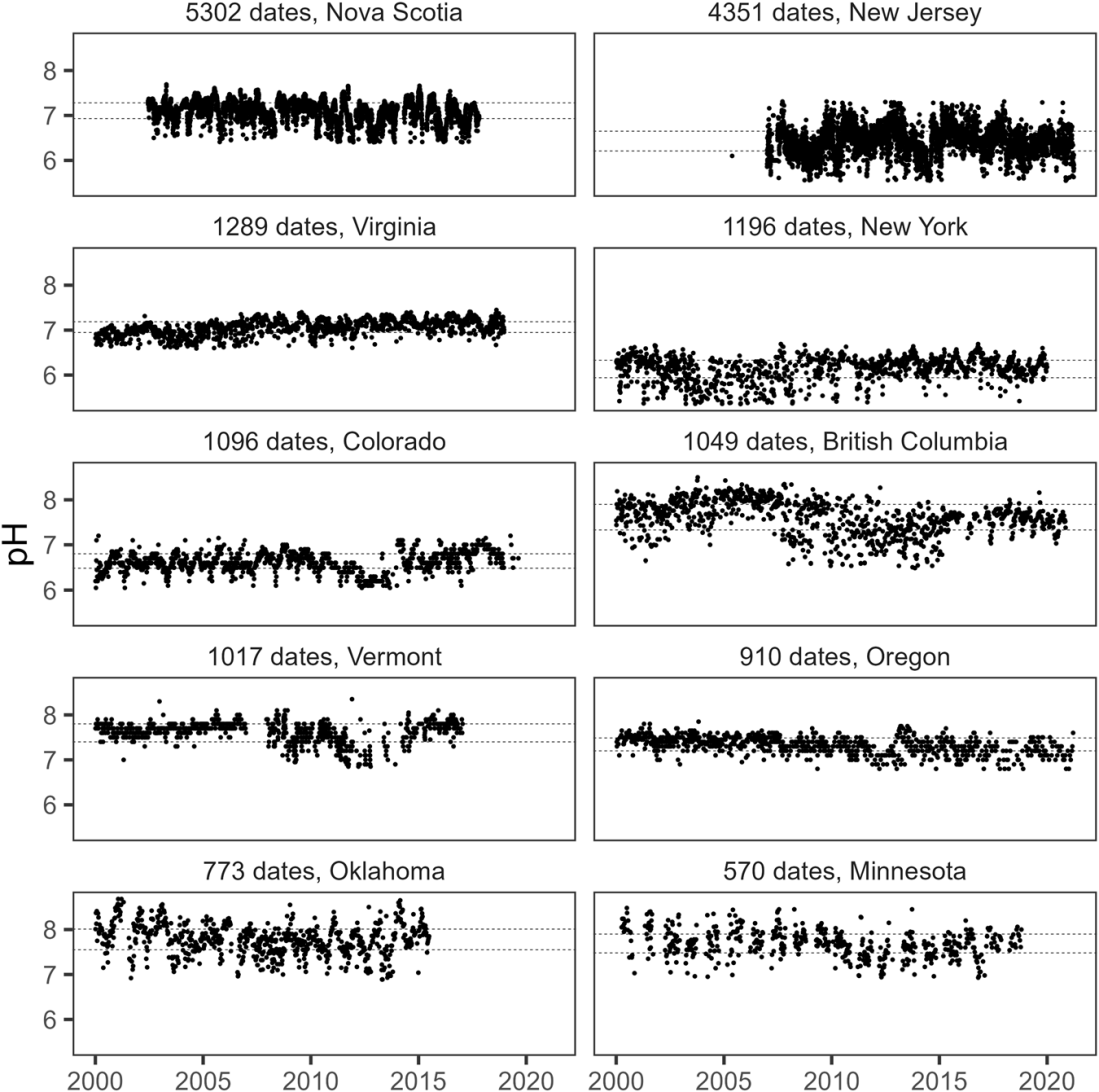
Temporal trends in pH for ten sites, selected (from among the 100 sites with data for the most dates) to have the longest temporal coverage and to come from 10 different administrative regions; source region given for each plot, along with number of dates with data. Dotted lines mark the interquartile range for each site. Outliers have been removed for presentation, but were not excluded from calculation of site statistics.

The final pH database used for the interpolations included records for 208,784 sites; the publicly shareable dataset includes 201,183 sites. The median pH across all sampled sites was 7.9, and 95% of sites had a median pH between 5.4 and 8.74 (Fig. 6). Density of sites was high across much of the USA, with a considerably higher number of sites than for calcium (Fig. 7a). Coverage tended to be sparser for Canada, with some areas, such as northern Saskatchewan and northern Manitoba, having fewer sites with available data compared to calcium. Compared to the calcium data, a greater proportion (67%) of sites had data from more than one date, and sites tended to have data from a greater number of sampling dates (median dates sampled = 4, mean dates sampled = 17.2). However, there were again areas of Quebec and Ontario where the data tended to be based on single values for each site (Fig. 7b). Temporal fluctuation at individual sites was also evident for pH (Fig. 8). However, the scale of temporal variation for individual sites was again smaller than the spatial variation among sites. The median interquartile range for pH measurements at individual sites was 0.3, with 75% of sites having an interquartile range of less than 0.46; the interquartile range across all sites (spatial variability) was 0.97.

Error metrics for the pH interpolations were generally very low, including RMSE and MAE; this is to be expected, since the restricted range of feasible pH values makes extremely large errors impossible. While it is not valid to directly compare most metrics between interpolations with different scales and based on different data, it is worth noting that the proportional errors (MSA) were considerably lower for the pH interpolations compared to the calcium interpolations. It is important to be aware, however, that since pH is measured on a logarithmic scale, apparently small differences may have comparatively large physical and chemical implications. There was little variation in the accuracy of the different interpolation methods, with most error metrics being similar for most of the methods (Table 9). However, OK-ZN had the best (or equal-best) scores in every metric excluding the proportional error, which was very close to the lowest value. For individual provinces, territories and states, the situation was similar (Tables 7, 8); OK-ZN was the best or equal-best method in 46 cases, with IDW-OM / IDW-OR being slightly better for the others. Consequently, the zero-nugget kriging interpolation (OK-ZN) was selected as the preferred interpolation method.

**Table 9:** Error Metrics for pH interpolation methods, generated via leave-one-out cross-validation.

Method	*r*	RMSE	MAE	MBE	MSA
NN	0.78	0.55	0.35	0.007	0.029
IDW-OR	**0.84**	0.46	**0.30**	0.007	**0.027**
IDW-OM	**0.84**	0.46	**0.30**	0.008	**0.027**
OK	0.81	0.49	0.33	**0.001**	0.031
OK-ZN	**0.84**	**0.45**	**0.30**	**0.001**	0.028

The best interpolation methods for each metric are highlighted in bold. Metrics: RMSE = Root Mean Square Error, MAE = Mean Absolute Error, MBE = Mean Bias Error, MSA = Median Symmetric Accuracy. Methods: NN = nearest neighbour; IDW-OR = inverse distance weighting, RMSE-optimised; IDW-OM = inverse distance weighting, MAE-optimised; OK = ordinary kriging, unconstrained nugget; OK-ZN = ordinary kriging, zero nugget.

**Fig. 9:**
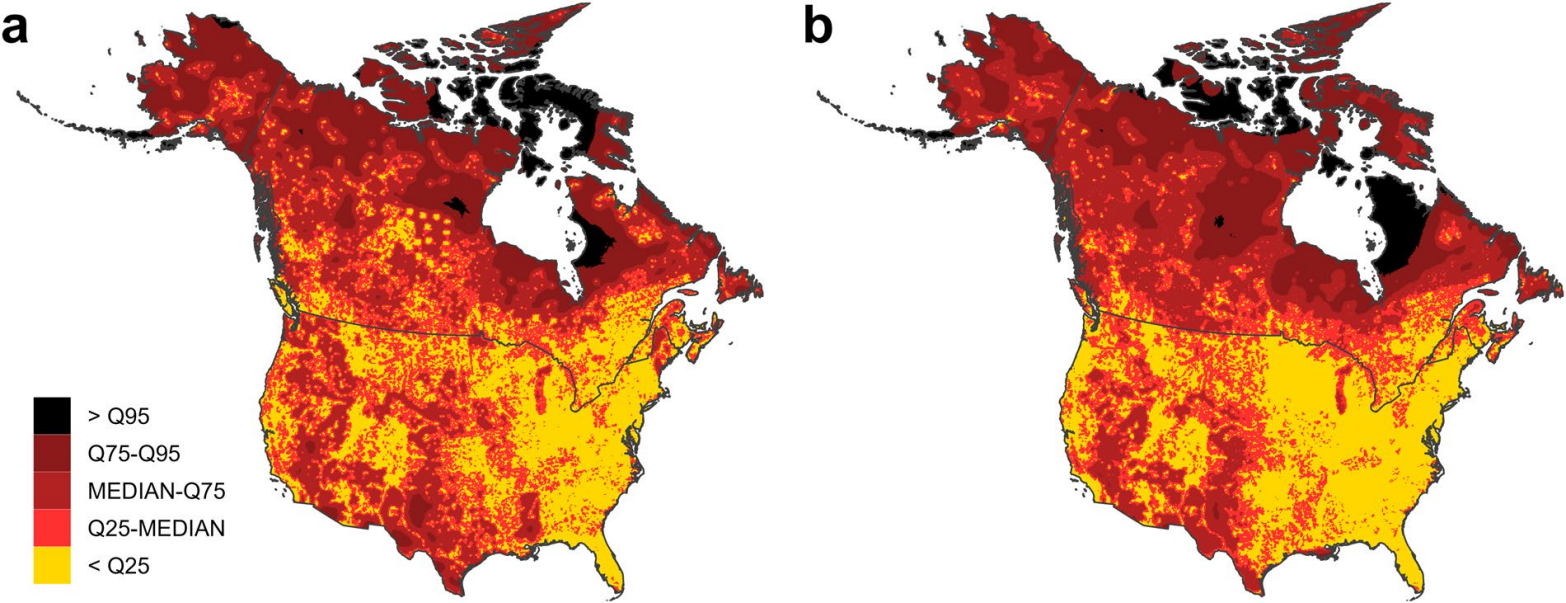
Kriging variance maps for (**a**) calcium and (**b**) pH interpolations. Q25, Q75, and Q95 are 25^th^, 75^th^, and 95^th^ percentile values.

Kriging variance maps generated by the selected interpolation methods can be used to identify areas of higher uncertainty in the interpolated values, and maps for the two variables show broadly similar patterns (Fig. 9). Across much of the USA and some of the Canadian provinces, there were high densities of sites (Figs. 4a, 7a); kriging variance was lower in these areas, indicating comparatively lower uncertainty in the interpolated values. Variance, and therefore uncertainty, was highest in the northern areas of the continent, where there were fewer sites with data. Interpolated values in such areas should be treated with some caution, since they are more distant from locations with measured values. These areas could be prioritised for future sampling if more certainty is required for estimates of pH and calcium concentrations.

## Usage Notes

### Example application – invasive species risk assessment for dreissenid mussels

To illustrate the advantages of high-resolution calcium and pH data, Wilcox *et al*.^143^ performed a continental-scale risk assessment for two species of invasive, freshwater dreissenid mussels using the new data layers. The two species, the zebra mussel *Dreissena polymorpha* and the quagga mussel *D. rostriformis bugensis*, have significant ecological and economic impacts on freshwater ecosystems in North America^144^, but can only survive, grow, and reproduce in waters with sufficiently high concentrations of dissolved calcium and within a particular pH range^5^.

Due to the lack of high-resolution calcium and pH data, previous risk assessments^13,15^ for these species have been limited to ecoregion- or sub-drainage-level resolution, and have not included Alaska and large areas of Canada (the Maritime provinces, Newfoundland and Labrador, and the Arctic). By combining the new calcium and pH data layers with additional high-resolution bioclimatic variables (e.g. temperature) from WorldClim^145^, Wilcox *et al*.^143^ were able to model habitat suitability for both dreissenid species for the entire extent of Canada and the continental USA, assess the importance of calcium and pH relative to additional bioclimatic drivers of mussel distributions, and calculate the relative risk of invasion for every Canadian province and territory at a 10 km^2^ resolution.

### Limitations

‘Big data’ approaches can be a useful tool for water quality projects, but are not without limitations^146^, and the aggregation of many different data sources, with highly variable quality control standards, necessitates some care in their use. Given the extremely large number of data points involved, inspection of individual data records was not possible. Therefore, some of the data filtering and cleaning approaches may have resulted in the exclusion of some valid records. On the other hand, it is likely that some low-quality data remain in the final database used for the interpolations. For example, while certain sources provided enough information to be able to quickly screen out inappropriate sample types, most sources did not. Points with large or obvious errors in their values or positions were easy to identify, and therefore to remove or correct. Records with incorrect or inaccurate – but plausible – values or positions were effectively impossible to identify and remove. The large number of records used for the final calcium and pH databases should, however, minimise the impact of these types of error on the final interpolations.

There are also a few limitations to using spatial interpolation to create such large-scale maps of water quality variables. Calcium concentrations and pH are primarily driven by the underlying geology^147^; transitions between underlying rock types can be relatively well delineated, and interpolation across such boundaries may produce results that do not reflect reality. This problem is likely to be minimised in areas with a high density of data points but may be important in data-poor regions. For example, there are relatively large swathes of northern Quebec and Arctic Canada for which no calcium or pH data were available. This may not be problematic in some contexts; for example, in the case of invasive species risk assessment, there are other factors (low temperature, remote location) that may make these areas low risk for many non-native organisms. Geological proxies could be used to predict calcium concentration in locations with no water quality data^148^, but this requires detailed geological information and validated mechanistic models and does not account for effects of plant cover and land use, which influence water chemistry^4,149^. Despite these limitations, geological data could be used to help improve predictions in areas with lower data coverage, or higher uncertainty, via co-kriging, which allows relationships with additional variables to be used during the interpolation process^150,151^. Alternatively, a range of machine learning approaches are available which are also able to use additional information, such as geological data and other environmental covariates; these methods can perform better than traditional geostatistical methods for generating spatial interpolations^152,153^, particularly when the density of data points for the primary variable of interest is low^154^. However, they do not always generate more accurate interpolations; a combined approach, which uses an ensemble of outputs from different interpolation methods with spatially-varying weightings dependant on density of available data, may result in better overall accuracy^136^. Such exercises are good candidates for future improvement to these data layers.

## Data Availability

The associated GitHub repository^142^ (https://github.com/andrew-guerin/water_quality_interpolations) contains code used to perform the final interpolations, scripts for reprojection and resampling of rasters, copies of the interpolated data layers, copies of the calcium and pH databases used for the interpolations (excluding proprietary data from third parties, which cannot be publicly shared under existing data agreements), and Shiny app scripts for interactive maps which show the distribution of data points, with summary data (where these can be shared). Please note that, as a result of the large number of data points included, the Shiny maps may take a few moments to load.
